# MITAS: A model for assessing the time-dependent risk of sequential applications of pesticides for soil organisms by consideration of exposure, degradation and mixture toxicity

**DOI:** 10.1016/j.mex.2019.12.004

**Published:** 2019-12-16

**Authors:** Alexandra Sybertz, Martina Roß-Nickoll, Andreas Schäffer, Björn Scholz-Starke, Benjamin Daniels, Richard Ottermanns

**Affiliations:** Department of Environmental Biology and Chemodynamics, Institute for Environmental Research, RWTH Aachen University, Germany

**Keywords:** MITAS – Mixture Toxicity of Application Spray series, Spray application, Mixture toxicity, Earthworm, Agricultural landscape, Environmental risk assessment

## Abstract

In agricultural landscapes it is common practice to apply pesticides as a spray series. Within a vegetation period multiple applications result in a mixture of different pesticides in the soil and other environmental compartments.

• A model named MITAS (MIxture Toxicity of Application Spray series) has been developed to calculate the soil concentration of pesticides and the resulting time-dependent mixture risk for earthworms. MITAS creates tables and graphs representing the mixture risk for an applied spray series time-dependently.
• A simulation of the impact of application series for a period of up to three years is possible with MITAS. Calculation of the soil concentration is based on the assumptions of the German pesticide registration. Mixture risk for each day within the entire modelling period is calculated.
• Mixtures of pesticides from former and multiple applications are the rule in agricultural soils as various studies have shown. Thus, considering the impact of pesticide mixtures is necessary to protect soil organisms. MITAS can assess not only the maximum mixture risk for soil organisms, but also how long a certain risk threshold may be exceeded, above which unacceptable effects on exposed organisms may occur.

## Graphical abstract

**Figure:**
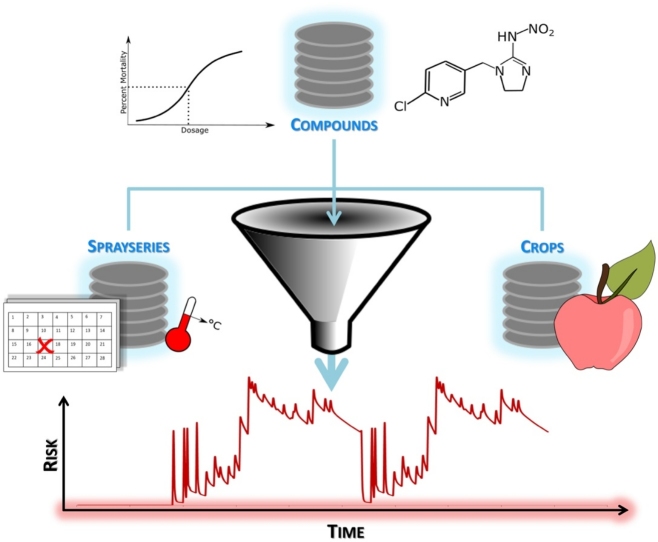

**Specification Table**

**Table:** 

Subject Area:	Environmental Science
More specific subject area:	Ecotoxicology
Method name:	MITAS – Mixture Toxicity of Application Spray series
Name and reference of original method:	*FOCUS soil persistence models [dataset] [13] and the calculation of the mixture risk with concentration addition [dataset] [17]*.
Resource availability:	*NA*

## Method details

### Modelling mixture toxicity with MITAS

Multiple applications of various pesticides in conventional agriculture result in multiple exposure of non-target organisms [dataset] [1]. The PAPA-survey (Panel crop protection applications) detected for example up to 20 applications of one or several pesticides in apple orchards [dataset] [2]. In such a scenario a mixture of pesticides can on the one hand be generated by joint use of several pesticides at the same time (tank mixture). On the other hand it can result from an application of single pesticides or pesticide mixtures (tank mixture, combination product) in a chronological time sequence (here defined as spray series), as some substances can remain for a long time in the ground based on their specific degradation time [dataset] [3]. Indeed, recent investigation detected a multitude of pesticide residues from former applications in agricultural soils [dataset] [[4], [5], [6]]. Therefore the consideration of mixtures is of utmost importance in ecotoxicological risk assessment, as a mixture can cause a significant effect even if the individual compounds of the mixture are contained in concentrations below their individual effect thresholds [dataset] [7]. The aim of our newly developed model MITAS (Mixture Toxicity of Application Spray Series) is the description and visualization of pesticide spray series and their time-dependent risk for soil organisms.

### Model structure

**Fig. 1:**
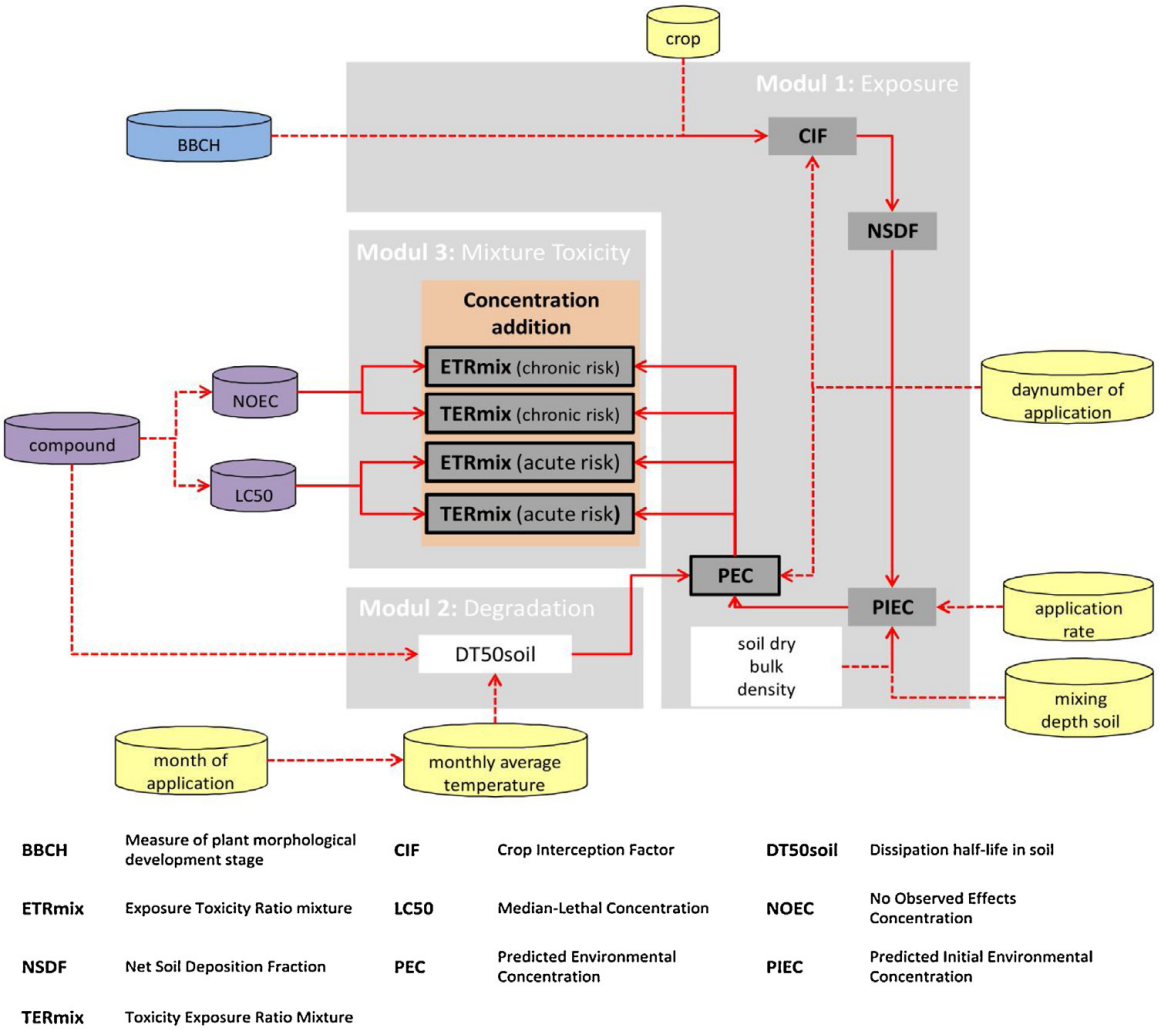
Main structures of the model MITAS. Cylinders represent input data taken from various internal databases. Input data are entered in the table maintab (yellow cylinders), whereas the table croptab (blue cylinder) includes information about the crop used. Information about applied substances is stored in the table compoundtab (purple cylinders). Mixture toxicity is calculated by concentration addition (orange box). Boxes with frames show output-data. Dotted arrows display information taken from the individual MITAS-databases.

MITAS predicts the time-dependent mixture risk of pesticide spray series. In general the model consists of three different modules, which consider exposure, degradation and mixture toxicity (Fig. 1).

MITAS is a script programmed in R [dataset] [8] which accesses the three data tables compoundtab, croptab and maintab. To run the R-script, the R package “ggplot2″ [dataset] [9] has to be installed and three data tables have to be stored locally. The command “dir.create()” generates the 4 folders ‘plots_PEC’, ‘plots_All’, ‘plots_TER’ and ‘plots_ETR’ automatically within the actual working directory of R-Studio.

To simulate an application scenario, maintab needs to be filled with data. Table 1 characterizes the variables used in maintab (yellow cylinders, Fig. 1). maintab contains important information concerning application-specific settings, regulatory assumptions and environmental variables, such as mixing depth or temperature. Any information of the application series has to be stored in maintab. An application series consists of single applied active substances with an application date (converted to julian day to obtain “day number”), an application rate in kg a.i./ha or the average monthly temperature of the year.

**Table 1:** Variables in maintab. User-dependent variables in the maintab of MITAS. All cells of the maintab have to be filled in before running the model.

variable	unit	description
description	/	User chosen name of the simulation
compound_name	/	Names of the substances applied in chronological order [Important for labelling the results tables].
Com_ID	/	The specific number of the active substances used. Regarding the Compoundtab, the Com_ID is the substance row number minus one.
numb_applications	/	Total number of applications in the application series. Every single substance at one date counts as one application.
application month	[MM]	The month of the application date.
ApplicationRate_kgai_ha	[kg a.i./ha]	The rate of the active ingredients which is applied at this application date.
application day	[julian day number]	The julian day number of the application date.
crop_id	/	Is specific for the treated crop and is stored in the croptab.
BBCH	/	The specific developmental stage of the crop which must be filled in for every single application.
degradation factor	/	Modifies the degradation halftime in soil. If the user enters 1 as degradation factor, the normal degradation halftime value from the compoundtab is used. Otherwise the degrdation factor is multiplied by the degradation halftime of the compoundtab.
mixing depth [m]	[m]	Is selectable as fixed value or as variable value. If the user writes "mix" into the cell the mixing depth varies between 2,5 and 1 cm depending on the kfoc-value. This variant is taken in the German pesticide registration.
days of year	[days]	A variable for the simulation time in days.
TEMP_JAN-DEC	[°C]	The average temperature for each month in the year.
year climate	[JJJJ]	If a year is inserted the related monthly average temperature data of Germany from the German Meterological Servic are used. If there is no year climate inserted the tool uses user specific information [TEMP_JAN-DEC]
CROP_CALENDAR_ REGION_ID	/	The crop calendar region of the FOCUS Scenario. This is no variable because only the FOCUS-Scenario for Hamburg (2) is implemented.

compoundtab (purple cylinders, Fig. 1) is the substance database used in MITAS that stores information about the individual compounds. It holds information about the physicochemical properties of the active ingredients and the ecotoxicological endpoints for risk assessment (Table 2).

**Table 2:** Input data in compoundtab. Table displays the compound specific data of the compoundtab.

data	unit	description
Compound ID	/	The specific number of the used active ingredients. Regarding the compoundtab the Compound ID is the substance row number minus one. Compound ID is the same as Com_ID in the maintab.
Compound name	/	The names of the applied substances in a chronological order [This names are important for the labelling in the result tables].
CAS nr	/	CAS (Chemical Abstracts Service) Registry Number
Chemical class	/	Optional: Is used if values are missing.
Chemical use	/	Optional: Is used if values are missing.
DegT50 soil	[d]	Dissipation halftime of the substance in soil.
LC50 earthworm	[mg ai/kg dw]	Median-Lethal Concentration
NOEC earthworm	[mg ai/kg dw]	No Observed Effect Concentration
K_foc_	/	Freundlich sorption constant normalised for organic carbon content

If input values for degradation or for toxicity are not available, they are estimated by MITAS. compoundtab stores the chemical class and agricultural use of the compounds. MITAS searches for all compounds with the same chemical class as the compound with the missing degradation or toxicity value which then is replaced by the average value of all compounds of the same chemical class. If no compounds of the same chemical class are available compounds with the same agricultural use are considered analogously. This principle is based on the missing value routine in HAIR2010 [dataset] [10].

### Exposure

MITAS calculates the crop interception factor (CIF) using the BBCH approach (German Federal Biological Research Centre for Agriculture and Forestry (BBA), German Federal Plant Variety Office and chemical industry) [dataset] [11]. The BBCH scale is a measure of the morphological developmental stage of a plant. Corresponding crop interception values of the BBCH-stages are defined by the FOCUS Groundwater Scenario report [dataset] [12]. Currently the interception calculations for 23 different crops for the FOCUS region Hamburg are implemented in MITAS. This information is contained in the table croptab (blue cylinders Fig. 1).

Combined with information about the used crop and the application date, a crop interception factor is calculated. Each substance is allocated a specific compound-ID based on their position in compoundtab.

### Prediction of soil concentration

The PIEC value represents the Predicted Initial Environmental Concentration of a pesticide directly after application. PIEC-values are calculated for each individual substance applied at each application date. The PIEC-calculation is based on the FOCUS soil persistence models [dataset] [13] (1), where AR is the application rate [g/ha], CIF is the crop interception factor, d is the mixing depth [cm], and p is the dry soil bulk density [g/cm^3^]. The resulting PIEC-value is in the unit mg/kg. A constant value of 1.5 g/cm^3^ is assumed for the dry soil bulk density on the basis of FOCUS soil persistence model [dataset] [13].

(1) $$PIEC_{soil}=\frac{AR*\left(1-CIF\right)}{100*d*p}$$

The mixing depth can be entered as fixed value or with the option “mix” as variable value. The option “mix” calculates a mixing depth of 1 cm for pesticides with K_foc_-values above 500 L/kg, otherwise a mixing depth of 2.5 cm is assumed [dataset] [14]. K_foc_-values >500 L/kg indicate high adsorption of the substance to the solid matrix and, thus a low leaching potential. A mixing depth of 1 cm leads to 2.5 higher initial exposure concentrations compared to a mixing depth of 2.5 cm.

### Substance degradation

First-order reaction kinetic is assumed to calculate substance degradation. The disappearance rate constant k is calculated in accordance to FOCUS soil persistence models [dataset] [13,15] (2), where k is the disappearance rate constant [d^−1^]; and DT50s is the modified dissipation half-life in soil [d]. The dissipation half-life is modified with a temperature correction factor based on the Arrhenius Eq. (3), where DT50s is the modified dissipation half-life in soil [d]; DT50soil is the degradation half-life in soil at reference temperature [d]; E_a_ is the average activation energy [54000 J/mol]; R is the molar gas constant [8.314 J/(mol*K)]; T_s_ is the reference temperature [K]; and T is the real temperature. Temperature specification is based on the user-dependent data in *maintab*. Temperature varies on a monthly basis.

(2) $$k=\frac{\ln(2)}{DT50_{s}}$$

(3) $$DT50_{s}=DT50_{soil}*\exp\left(\left(\frac{E_{a}}{R}\right)*\left(\frac{1}{T_{s}}-\frac{1}{T}\right)\right)$$

### Mixture risk

The individual risk Exposure Toxicity Ratio (ETR) is calculated as quotient of predicted environmental concentration (PEC) and the ecotoxicological endpoint [dataset] [16]. The reciprocal risk value, Toxicity Exposure Ratio (TER), is also determined. For acute risk the ecotoxicological endpoint is Median-Lethal Concentration (LC50, OECD207) (up to now only for earthworms). No Observed Effects Concentration (NOEC, OECD222) is used as chronic endpoint (up to now only for earthworms). Each simulation day is treated as a single pesticide mixture consisting of the concentration of each substance in soil at that specific day, regardless whether residues of previously or freshly applied substances are considered.

Calculation of mixture risk in MITAS is based on the concept of concentration addition with the aspect of multi-component mixtures [dataset] [17,18] (4), where n is the number of components, i is the substance, c is the substance concentration in the mixture, and ECx is the effect concentration of the substance.

(4) $$\sum_{i=1}^{n}\frac{c_{i}}{ECx_{i}}=1$$

Warne [dataset] [19] stated that about 70 % of the mixtures act in conformity with the prediction of concentration addition. This confirms the funnel hypothesis which predicates that an increasing number of components in a mixture leads to an increasing tendency to act similar to the concentration addition (CA) [dataset] [20].

To obtain the mixture risk index ETRmix (Exposure Toxicity Ratio Mixture) based on the concentration addition approach ETR-values of each compound of a mixture are summed up on a daily basis [dataset] [21]. Thereby the time-dependent mixture risk is obtained. Calculation of acute and chronic risk for each individual day is possible in MITAS, resulting in acute and chronic ETRmix values.

TERmix (Toxicity Exposure Ratio Mixture) calculation is based on concentration addition (CA) [dataset] [22] (5), where TER(mix) is the TER-value of the mixture, i is the individual mixture component, and TER(a.s.) is the TER-value of the individual mixture components.

(5) $$TER(mix)={\left(\sum_{i}\frac{1}{TER\left(a.s._{i}\right)}\right)}^{-1}$$

### Presentation of simulation results

**Fig. 2:**
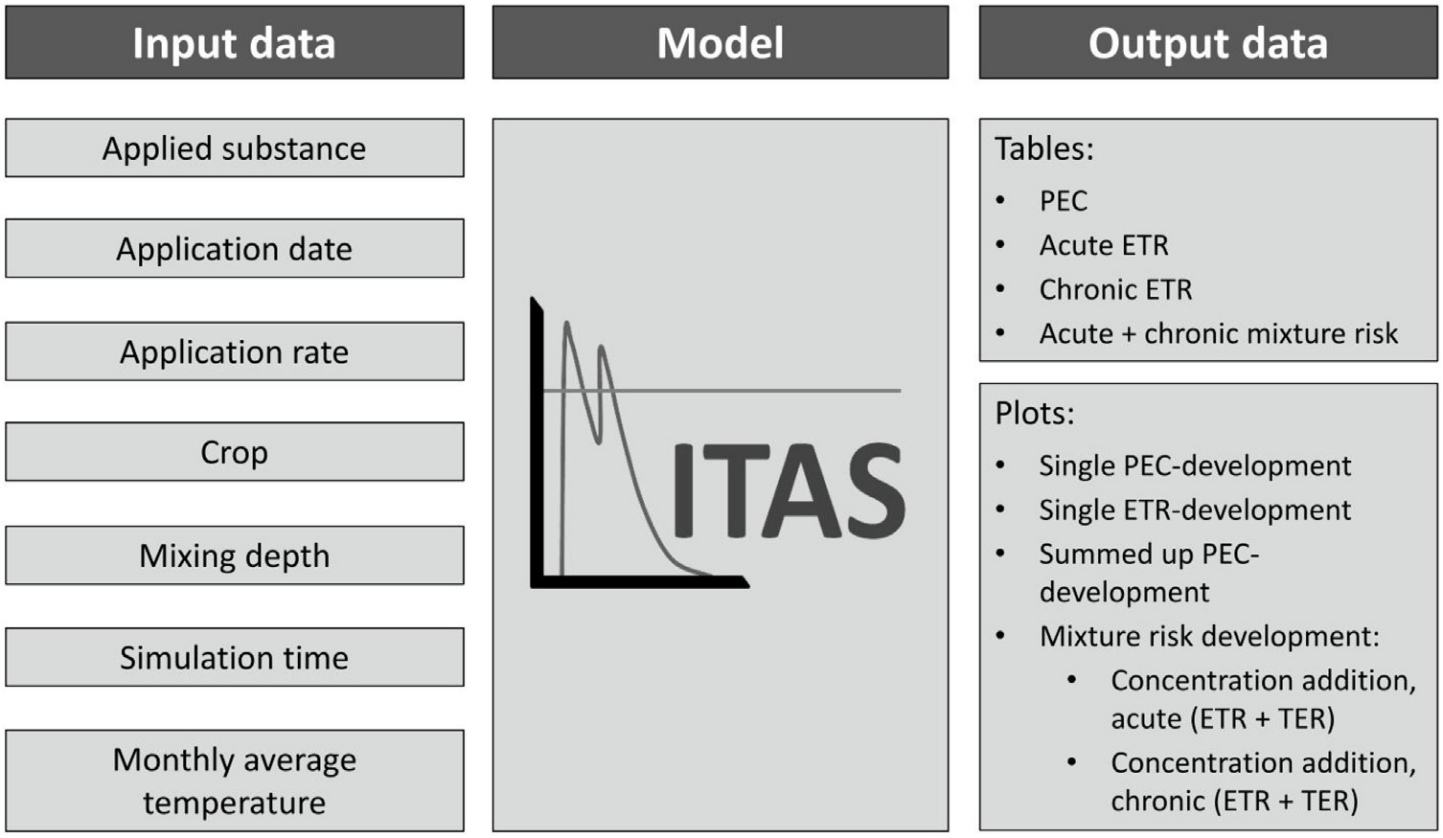
Input and output data of MITAS.

The simulation results, consisting of csv-files and plots, are automatically stored in different folders (Fig. 2). The individual PEC- and ETR-courses of each substance are stored in the folders ‘plots_PEC’ and ‘plots_ETR’. A folder named ‘plots_All’ comprises plots of the overall risk in consideration of mixture toxicity (ETRmix). Results of mixture risk calculated from TER-values (TERmix) are stored separately in the folder ‘plots_TER’ (Fig. 2).

### Application example

An example calculation shows which results are generated in MITAS. A pesticide spray sequence in apple was simulated. Physicochemical and toxicological substance data of the example spray series were taken from the PPDB (Pesticides Properties Database) [dataset] [23].

**Fig. 3:**
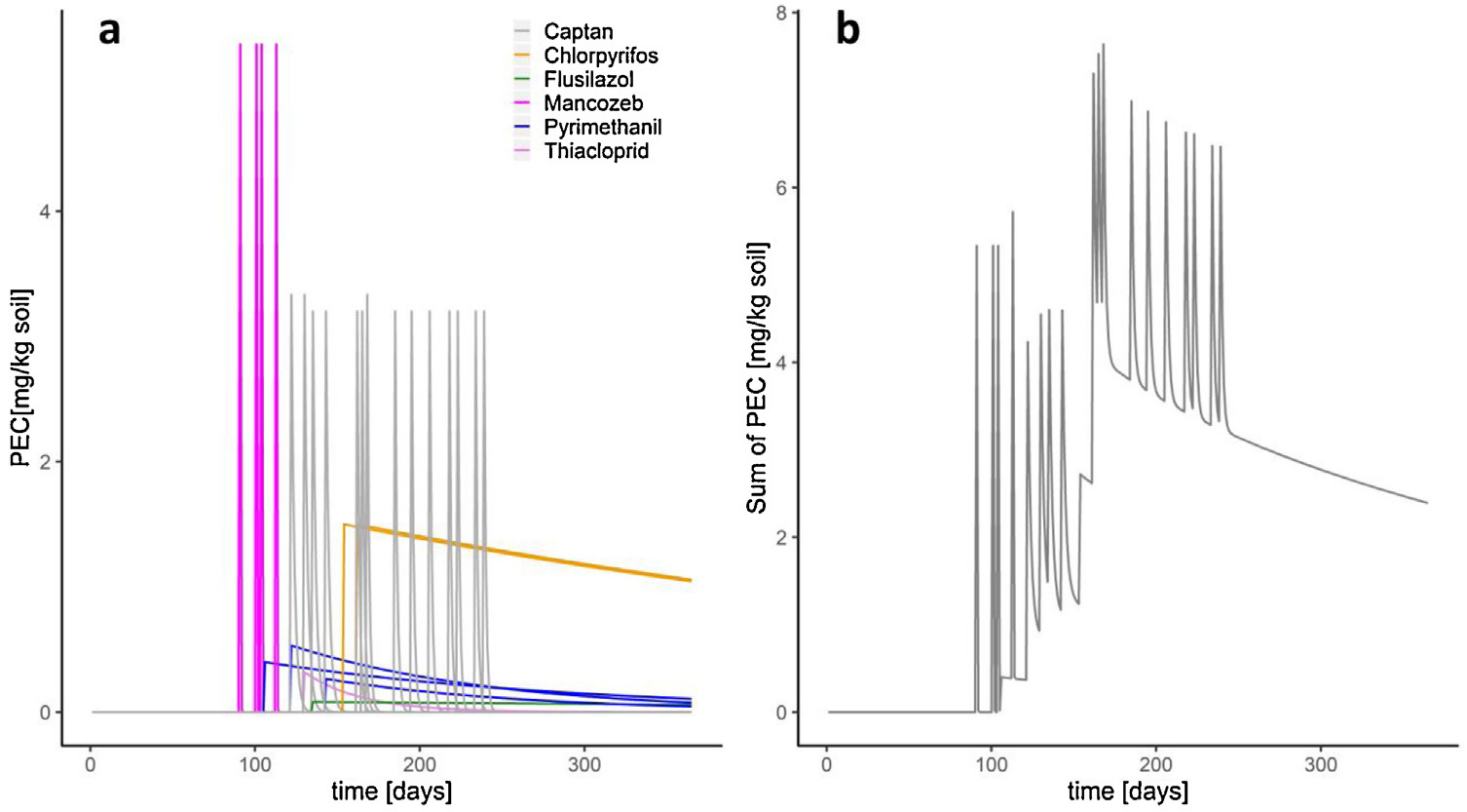
PEC predictions of the individual pesticides of a spray series (a) and the cumulated PEC using MITAS (b). The x-axis represents single days of a year, the y-axis PEC-values. The degradation of the substances over time is taken into account. Different colors in the left scheme (a) were used for the individual substances.

MITAS first calculates and visualizes the individual PECs for each substance and then the cumulated PEC values for all substances applied (Table 3, Fig. 3). The calculated PEC values serve as the basis for the calculation of the mixture risk (ETR, TER).

**Table 3:** Extract from an MITAS-table in which the time-dependent PEC values for each individual pesticide are stored. Each substance applied is displayed in one column in chronological order. Same substances applied at different dates are listed separately.

days	Mancozeb	Mancozeb	Mancozeb	Pyrimethanil	Mancozeb	Pyrimethanil
1	0	0	0	0	0	0
2	0	0	0	0	0	0
…	…	…	…	…	…	…
90	0	0	0	0	0	0
91	5.33333333	0	0	0	0	0
92	0.01952397	0	0	0	0	0
93	7.15E-05	0	0	0	0	0
94	2.62E-07	0	0	0	0	0
95	9.58E-10	0	0	0	0	0
96	3.51E-12	0	0	0	0	0
97	1.28E-14	0	0	0	0	0
98	4.70E-17	0	0	0	0	0
99	1.72E-19	0	0	0	0	0
100	6.30E-22	0	0	0	0	0
101	2.31E-24	5.33333333	0	0	0	0
102	8.44E-27	0.01952397	0	0	0	0
103	3.09E-29	7.15E-05	0	0	0	0
104	1.13E-31	2.62E-07	5.33333333	0	0	0
105	4.14E-34	9.58E-10	0.01952397	0	0	0
106	1.52E-36	3.51E-12	7.15E-05	0.4	0	0
107	5.55E-39	1.28E-14	2.62E-07	0.39796516	0	0
108	2.03E-41	4.70E-17	9.58E-10	0.39594067	0	0
109	7.43E-44	1.72E-19	3.51E-12	0.39392648	0	0
110	2.72E-46	6.30E-22	1.28E-14	0.39192254	0	0
111	9.96E-49	2.31E-24	4.70E-17	0.38992879	0	0
112	3.65E-51	8.44E-27	1.72E-19	0.38794519	0	0
113	1.34E-53	3.09E-29	6.30E-22	0.38597167	5.33333333	0
114	4.89E-56	1.13E-31	2.31E-24	0.3840082	0.01952397	0

**Fig. 4:**
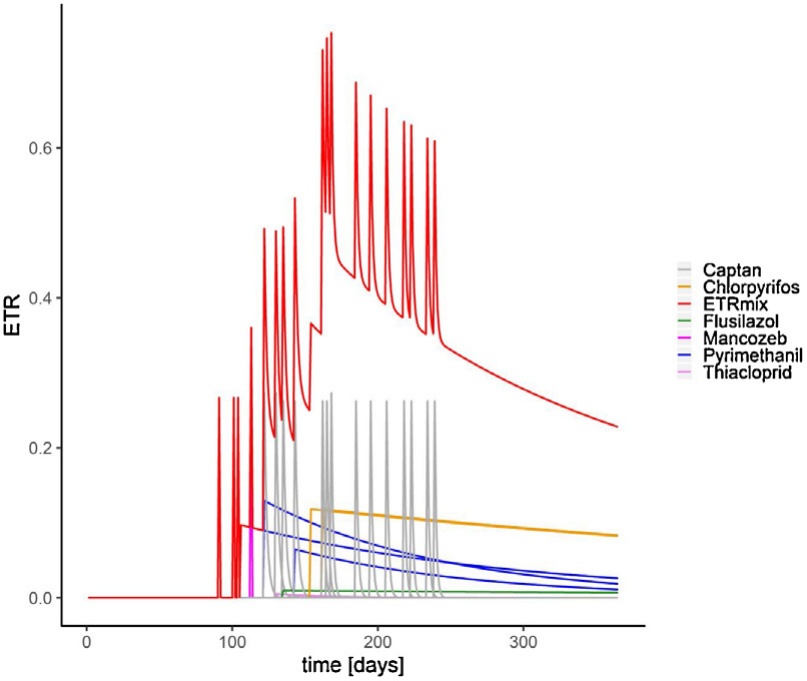
Prediction of the single and overall chronic risk development of a spray series using MITAS. The x-axis represents single days of a year, the y-axis ETR values. The red line (top line) represents the chronic mixture risk (ETRmix) calculated with concentration addition and NOECs from earthworm reproduction studies, whereas the differently colored lines underneath represent the individual applied substances. The degradation of the substances over time is taken into account.

The acute and chronic mixture risk can be presented as ETRmix as well as the reciprocal unit TERmix. In Fig. 4 the time-dependent chronic risk of the pesticide mixture, ETRmix, and that of the individual substances is shown. It is obvious that the maximum mixture risk (red line) is higher than the maximum risk of the individual substances (magenta line, mancozeb). Also, it is important to note that the mixture risk remains at a high level over a long period of time and, in fact, does not reach the zero level until end of the simulation (one year).

**Fig. 5:**
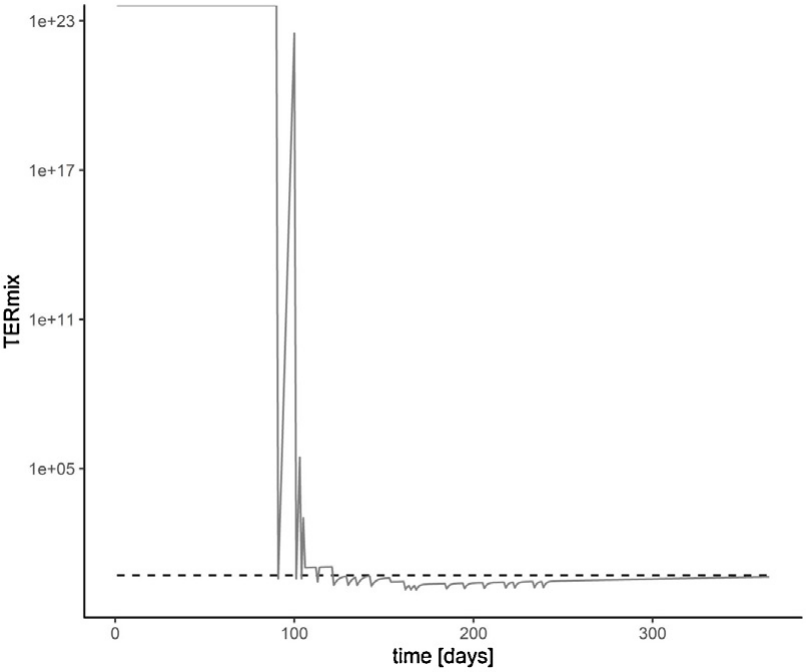
Prediction of the overall chronic risk development of a spray series using MITAS. The x-axis represents single days of a year, the y-axis TERmix values calculated with concentration addition. The threshold value for chronic risk is represented by the dotted line.

The lower the ratio of toxicity and exposure (TER), the higher is the risk. Corresponding threshold values are set by the European Commission [dataset] [24,25]. The plot generated by MITAS compares the predicted chronic mixture risk (TERmix) with the threshold value for chronic risk (Fig. 5). The TER values are represented using a logarithmic scale, since the TER value can become infinitely large with very small PEC values. In the example spray sequence simulated here, TERmix falls below the threshold from day 122 onwards for a long period of time indicating an unacceptable risk for the exposed organisms (regarding the endpoint “reproduction of earthworms”).

### Model comparison

Three further models considering the fate and effects of pesticides are compared to MITAS in the following with a focus on the mixture risk. The models are (1) PRIME beta (ipmPRIME), (2) SYNOPS-WEB (JKI), (3) HAIR 2014 (HAIR). The spectrum of the performances of the three models is summarized in Table 4.

**Table 4:** General comparison of the tools PRIMEbeta, HAIR2014, SYNOPS-WEB and MITAS.

PRIMEbeta is the abbreviation for Pesticide Risk Mitigation Engine [dataset] [26]. The PRIME project started in 2008 for developing an online relative risk ranking tool [dataset] [27] for people who use pesticides, such as farmers. Our comparison refers to the PRIMEbeta tool of the Oregon State University Integrated Plant Protection Center. In PRIMEbeta each individual application of a pesticide is considered as a single independent event [dataset] [28]. The tool illustrates the risk of each substance applied and the cumulative risk for each endpoint assuming that at least one of the applications has an adverse effect [dataset] [28]. The task of the PRIME-project is to demonstrate the benefits of Integrated Pest Management (IPM) (IPM PRIME Mission Statement).

SYNOPS-WEB (*Synoptische Bewertung des Risikopotentials chemischer Pflanzenschutzmittel*, version 1.0, Synoptic assessment of the risk potential of chemical plant protection products) is developed by the Julius Kühn-Institute (JKI) Germany [dataset] [29]. The objective is to include more mitigation factors to assist the farmers in reducing the environmental risk. To calculate a soil risk score, the tool predicts time-dependent exposure curves and also includes degradation including the dependence on the temperature. The calculation of the chronic risk of an application series is based on the concentration addition concept. To estimate the chronic exposure the time-weighted average of exposure is calculated for seven days and each substance. The time-weighted concentrations are added on a daily basis and the highest mixture risk on a certain time point is defined as the overall chronic risk [dataset] [30].

At the research institute Alterra Wageningen a tool called HAIR2014 (Harmonised environmental Indicators for pesticide Risk) was developed to assesses the effectiveness of EU sustainable agriculture policy [dataset] [31]. The calculation of risk indicators is based on the HAIR consortium within the framework of the 6th Environmental Action Programme (Contract No. SSP-CT-2003-501997). Beside aquatic and terrestrial endpoints also endpoints for human risk are included [dataset] [32]. Pesticide concentrations in soil after multiple applications are defined as the exposure after the last application, taking into account residues from previous applications [dataset] [10]. This only applies to several applications of the same substance.

In the model PRIME the degradation of pesticides is only marginally taken into account. HAIR2014 is a transparent tool to predict the risk of one pesticide, but the tool does not consider mixture risk of an application series. HAIR2014 and SYNOPS-WEB calculate risk indicators for three different environmental compartments (soil, surface waters and field margins). Currently, our model MITAS calculates risk indicators only for soil. Long-term risk simulation for more than one year is possible in MITAS and HAIR2014, whereas SYNOPS-WEB has a fixed simulation time of one year. With the exception of PRIMEbeta, the calculated comparison value in the models is the ETR. MITAS furthermore calculates the TER value. Only MITAS calculates and visualizes the time-dependent overall risk and illustrates the period during which a risk threshold is exceeded (Table 4).

## Summary

The repeated use of pesticides as a spray series is widespread practice [2] resulting in pesticide mixtures in soil [5,6] and organisms [33]. Soil organisms, such as earthworms, are directly exposed to the application of pesticides. Due to the high number of authorized pesticides not all possible mixture combinations can be covered by ecotoxicological tests aiming to investigate effects on soil organisms. Therefore, model MITAS was developed to estimate the time-dependent exposure and risk of pesticide spray series on earthworms.

MITAS allows to readily and transparently evaluating the fate and impact of multiple pesticides applied in spray series and includes important aspects to predict the potential mixture risk for soil organisms (Table 5). Additionally to other already existing pesticide risk models, MITAS calculates and visualizes the time-dependent risks and depicts exceedances of harmonized thresholds (Fig. 5, Table 4). Navarro et al. [dataset] [34] highlighted the following aspects as particularly important to predict the potential environmental risk of pesticides: the chemical-physical properties, toxicity, mobility, and persistence of the compound, the application rate, the type of formulation, the method and time of application.

**Table 5:** MITAS summary.

√	Transparent calculation
√	Consideration of three phenomena: tank mixture, combination product & application series
√	Consideration of substance degradation
√	Time-dependent mixture risk (prediction & visualization)
√	Calculation possible for more than one year (influence of previous applications)
√	Risk endpoints: acute & chronic, ETR- & TER-values
√	Allows individualization (soil depth, simulation time, ecotoxicological input data)

MITAS considers not all but many of these aspects. Chemical-physical and ecotoxicological properties, and the degradation time of the toxic compounds are included as well as the application rate and time of application. In addition, interception by plants is considered to estimate the exposure of organisms. MITAS assumes always a liquid formulation type which can be applied through spraying.

To summarize, MITAS includes the most important parameters to predict the time-dependent pesticide mixture risk with a manageable amount of uncertainties. Currently, MITAS results are not yet validated with measured concentrations in soil. However, most exposure and fate assumptions in MITAS are based on the generally accepted assumptions of German pesticide registration. Based on Beck et al. [dataset] [35] “a “good” model should contain relatively few parameters yet be able to predict behavior accurately over a wide range of conditions”. We intend to further improve MITAS by, for example, including other terrestrial organisms and estimating the effects of pesticide spray series on populations.

## Declaration of Competing Interest

The authors declare that they have no known competing financial interests or personal relationships that could have appeared to influence the work reported in this paper.
